# Multiple comparisons

**DOI:** 10.1186/s12915-015-0199-0

**Published:** 2015-10-23

**Authors:** Emma Saxon

**Affiliations:** BMC Biology, BioMed Central, 236 Gray’s Inn Road, London, WC1X 8HB UK

## Abstract

Oat plants grown at an agricultural research facility produce higher yields in Field 1 than in Field 2, under well fertilised conditions and with similar weather exposure; all oat plants in both fields are healthy and show no sign of disease. In this study, the authors hypothesised that the soil microbial community might be different in each field, and these differences might explain the difference in oat plant growth. They carried out a metagenomic analysis of the 16 s ribosomal ‘signature’ sequences from bacteria in 50 randomly located soil samples in each field to determine the composition of the bacterial community. The study identified >1000 species, most of which were present in both fields. The authors identified two plant growth-promoting species that were significantly reduced in soil from Field 2 (Student’s *t*-test *P* < 0.05), and concluded that these species might have contributed to reduced yield.

## Comment

**Fig. 1:**
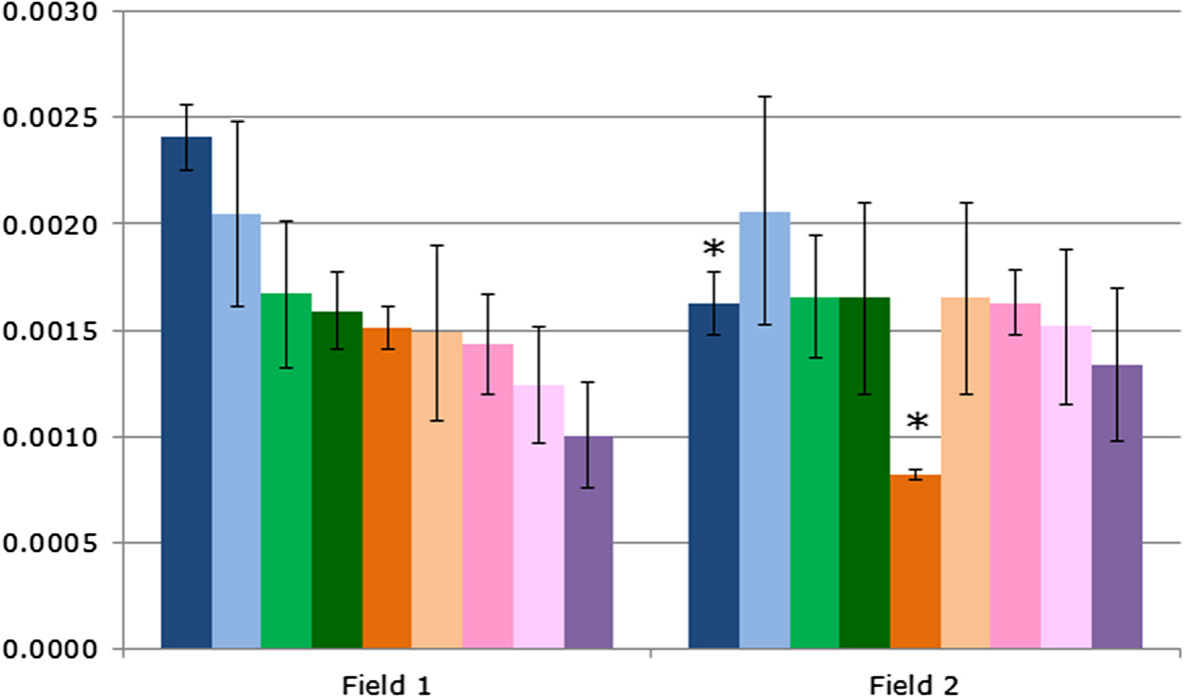
The proportion of nine known plant growth-promoting bacterial species detected in the soil bacterial community of two fields. Oat plant yield was greater in Field 1 than Field 2; two of the growth-promoting bacterial species were found at a significantly lower level in Field 2 than Field 1 (Student’s *t*-test *P < 0.05; error bars show standard deviation)

High-throughput genomic studies produce large amounts of data with the potential to be mined for information about gene regulation, evolutionary relationships, the genetic components of disease and, in this case, the composition of microbial communities in different environments. However, researchers encounter several potential pitfalls when analysing ‘big data’ that may lead to false conclusions. One of these is a problem of multiple comparisons; in this example, the authors compared the levels of the >1000 bacterial species found in two different fields, many of which were found to differ significantly between the two fields (Student’s *t*-test *P* < 0.05). The researchers focussed on the nine known plant growth-promoting species that were identified in their study (Fig. 1) in which the levels of two species were found to be significantly decreased, leading to the hypothesis that reduced plant growth-promoting species in Field 2 may have contributed to reduced oat yields.

However, significance at the level of *P* < 0.05 in a Student’s *t*-test means that the false positive rate — incorrectly rejecting the null hypothesis that there is no difference between the groups — is 5 %. When the number of comparisons is large, the likelihood of false positive results is greater: for the 1000 comparisons in this study, around 50 are expected falsely to show a significant increase or decrease in level. The researchers in this case did not correct for multiple comparisons, but several methods exist for doing so. The simplest of these is the Bonferroni correction, which uses a *P* value calculated as 0.05/n, where n is the number of comparisons made. But this method can ‘over-correct’, leading to significant differences being overlooked; Nakagawa’s 2004 review provides some more detail on this subject [1]. More complex methods, such as the Benjamini-Hochberg procedure, allow for a slightly less strict correction, reducing the rate of false negative results that occur with the Bonferroni method [2]. This is particularly useful when the number of comparisons is very large, as is the case in this study.
